# Occurrence and Dietary Exposure Assessment of Patulin and Major *Alternaria* Toxins in Commercial Infant Fruit Purées from Italian and Spanish Markets Using Targeted Analysis and Suspect Screening

**DOI:** 10.3390/toxins18100413

**Published:** 2026-09-24

**Authors:** Enrico Pambianco, Octavian Augustin Mihalache, Chiara Dall’Asta, Antonia Garrido Frenich, Laura Carbonell-Rozas

**Affiliations:** 1 Department of Food and Drug, University of Parma, Parco Area delle Scienze 27/A, 43124 Parma, Italy; pambiancoenrico08@gmail.com (E.P.); chiara.dallasta@unipr.it (C.D.); 2 Department of Chemistry and Physics, University of Almeria, Carretera de Sacramento s/n, 04120 Almeria, Spain; agarrido@ual.es; 3 R&D Department, Tentamus Iberia, Unit B-7, Calle Árbol del Amor, s/n, 04007 Almeria, Spain

**Keywords:** patulin, *Alternaria* toxins, mycotoxins, fruit purées, infant food, UHPLC-MS/MS, HRMS suspect screening, exposure assessment, risk characterization

## Abstract

The aim of this study was to evaluate the co-occurrence and to assess the dietary health risk of multiple mycotoxins in fruit-based purées intended for infant consumption, combining targeted quantification and dietary exposure assessment with high-resolution suspect screening. Forty samples with varying apple content were randomly collected from Italian (*n* = 20) and Spanish markets (*n* = 20). A fit-for-purpose ultra-high-performance liquid chromatography coupled to tandem mass spectrometry (UHPLC-MS/MS) method was developed and validated for the simultaneous determination of patulin (PAT) and three *Alternaria* toxins, including alternariol (AOH), alternariol monomethyl ether (AME), and tentoxin (TEN), providing satisfactory sensitivity, accuracy and precision. Targeted analysis revealed frequent co-occurrence of PAT, AOH, and AME, whereas TEN was not detected. Dietary exposure assessment indicated that PAT posed negligible health risks, whereas AOH and AME exceeded their respective Thresholds of Toxicological Concern (TTCs) across all evaluated population groups, indicating *Alternaria* toxins as the main contributors to dietary risk. High-resolution mass spectrometry (HRMS) suspect screening expanded the mycotoxin profile through the identification of tenuazonic acid, the tentative identification of altenusin and aflatoxin G2, and the annotation of 2,3-dihydroxybenzoic acid, a potential interferent in PAT determination. Overall, the proposed workflow provides a comprehensive strategy for mycotoxin monitoring in infant fruit purées and supports enhanced surveillance of emerging *Alternaria* toxins.

## 1. Introduction

Fruits and fruit-derived products constitute an essential component of a healthy diet and are widely recommended because of their contribution to the intake of vitamins, minerals, fiber and physiologically active compounds [1,2]. Given the high perishability of fresh fruits and the increasing consumer demand for versatile, palatable, nutritious foods, fruit processing has become an important strategy for extending shelf life, reducing post-harvest losses and broadening fruit consumption opportunities [2]. Among processed fruit products, fruit purées and compotes are widely consumed by infants and young children and often represent staple foods in their diet.

Mycotoxins are toxic secondary metabolites produced by filamentous fungi, mainly species of the genera *Aspergillus*, *Penicillium*, *Fusarium* and *Alternaria*, which can contaminate a wide range of crops, during the pre- and post-harvest stages [3,4]. Despite the technological treatments applied during processing, mycotoxins originating from infected raw materials may persist in fruit purées and compotes, as these compounds are generally stable and not fully eliminated by conventional processing steps [5]. This potential dietary exposure is of particular relevance for infants and young children, who exhibit higher food intake relative to body weight, immature detoxification systems and limited dietary diversity [1,6]. Such exposure has been associated with a range of acute and chronic adverse health effects, including gastrointestinal disorders, immunosuppression, endocrine-disrupting activity, carcinogenesis as well as neurological and developmental delays in infants and young children [1,7].

Among the mycotoxins relevant to fruit and fruit-derived products, patulin (PAT) is the most extensively studied; it is produced mainly by *Penicillium expansum*, the causal agent of blue mould rot in pome fruits and is widely regarded as a quality and safety indicator for apples and apple-based products [8,9]. In recent years, *Alternaria* toxins have attracted increasing attention as emerging contaminants of fruit and vegetable commodities [10,11]. *Alternaria alternata* produces a large number of secondary metabolites, among which alternariol (AOH), alternariol monomethyl ether (AME), tenuazonic acid (TeA) and tentoxin (TEN) are the most frequently reported in foods [12,13].

Beyond apples, other fruit species differ in their susceptibility to fungal colonization depending on the physiological and structural characteristics of the fruit surface. Grapes, for instance, are particularly prone to skin injuries that favor fungal entry and represent a matrix in which ochratoxin A (OTA) and fumonisins (FB1, FB2) have been reported [14,15]. In addition, several mycotoxins may undergo plant or fungal biotransformation into modified, or “masked”, forms such as glycosylated or sulphated conjugates, which retain toxicological relevance but generally elude routine targeted analysis [16]. *Alternaria* toxins are of particular concern in this respect, as several conjugated forms have been reported in fruits, like apple, alongside their parent compounds [17,18,19]. Moreover, *Alternaria* toxins are typically produced simultaneously by the same fungal strains, and their combined exposure may result in additive or synergistic toxic effects [12,20]. From a regulatory standpoint, PAT is the only mycotoxin with a harmonized maximum level in the European Union, set at 10 µg/kg in fruit-based foods intended for infants and young children by Regulation (EU) 2023/915 [21]. By contrast, *Alternaria* toxins are not yet formally regulated: Commission Recommendation (EU) 2022/553 provides only indicative levels for AOH, AME and TeA in a limited number of food categories, and fruit-based products for infants are not included [20,22]. This monitoring gap, combined with the projected influence of climate change on the distribution and co-occurrence of these toxins in Mediterranean areas, highlights the need for reliable multi-analyte occurrence data [23,24].

Liquid chromatography coupled with tandem mass spectrometry (LC-MS/MS), and in particular triple-quadrupole instruments operated in multiple reaction monitoring (MRM) mode, remains the reference approach for the sensitive and selective quantification of target mycotoxins in complex food matrices [25,26,27]. Targeted methods, however, are by definition restricted to a predefined set of analytes and cannot capture unexpected or emerging contaminants [28]. High-resolution mass spectrometry (HRMS), typically based on Orbitrap or quadrupole time-of-flight instruments, allows a complementary suspect-screening approach capable of covering a much broader range of fungal metabolites and their modified forms, thereby providing a more comprehensive picture of the mycotoxin profile of a given matrix [25,28,29]. Despite the availability of both approaches, occurrence and dietary exposure data on multiple mycotoxins in infant fruit purées remain limited [30], and most published studies focus on PAT alone or do not integrate suspect-screening strategies to explore the broader mycotoxin profile of these products [25,31,32,33].

The aim of the present study was therefore to assess, through targeted ultra-high-performance liquid chromatography coupled to tandem mass spectrometry (UHPLC-MS/MS), the occurrence of PAT and three *Alternaria* toxins (AOH, AME and TEN) in commercial fruit purées intended for infants and young children marketed in Italy and Spain, to complement this quantitative dataset with HRMS suspect screening for a broader panel of fungal metabolites and their modified forms, and to perform a dietary exposure assessment especially for the vulnerable population of infants and young children based on the targeted quantitative data.

## 2. Results and Discussion

Sample preparation is a critical step in multi-mycotoxin analysis, particularly for complex matrices such as fruit purées, where an efficient and selective procedure is essential to ensure accurate quantification of the targeted analytes [15,34]. In this regard, a fit-for-purpose ultrasound-assisted extraction (UAE) combined with a dispersive solid-phase extraction (d-SPE) sample treatment procedure was developed and optimized to maximize the recovery of the target mycotoxins while minimizing matrix effects prior to UHPLC-MS/MS analysis. The optimization of the proposed method was performed following a univariate approach. In the case of the sample treatment, the principles of Green Sample Preparation [35] were applied through the miniaturization of sample and solvent volumes, the omission of unnecessary reagents, the minimization of sorbent use, the optimization of sonication time, and the use of d-SPE clean-up instead of conventional SPE to reduce solvent consumption and processing time. Overall, these modifications contributed to a faster, simpler, and more sustainable analytical workflow.

### 2.1. Optimization of MS/MS Detection

Mass-spectrometric parameters were first optimized for each analyte by direct infusion of the individual standards at a flow rate of 0.4 mL/min. The precursor ion and the optimal ionization polarity were selected in full-scan mode by varying the fragmentor voltage, and the product ions were subsequently generated in MRM mode by tuning the collision energy. PAT, AOH, AME and TeA gave the most intense response in negative ionization mode, whereas TEN was detected in positive mode. For each compound, the most abundant transition was used for quantification and two further transitions for confirmation, in line with the minimum identification requirement of two product ions specified for MS/MS techniques [36]. For example, for PAT, the deprotonated molecule at *m*/*z* 153.0 produced the characteristic fragments at *m*/*z* 109.1 (quantifier), 81.0 and 53.2 (qualifiers). All the MS/MS conditions, including ionization mode, precursor/product transitions, fragmentor voltages and collision energies, for each analyte are summarized in Table S1 (Section 4).

### 2.2. Optimization of the Chromatographic Separation

The mobile phase and gradient elution method were selected based on evidence reported in previous studies and the ionization characteristics of the targeted analytes [33,37,38]. Acetonitrile was preferred over methanol owing to its higher ionization efficiency and better chromatographic resolution [37,39]. Acetic acid was added to both mobile phases, since patulin requires a slightly acidic environment to ensure its stability and satisfactory recovery [32,33]. Ammonium acetate was included in the aqueous phase instead of formic acid to reduce matrix effects and improve signal stability in both negative and positive ion ESI modes [38]. Gradient elution was therefore carried out with water containing 0.05% acetic acid and 5 mM ammonium acetate (solvent A) and acetonitrile containing 0.05% acetic acid (solvent B). The total run time was 12 min, including column re-equilibration, with an initial flow rate set at 0.3 mL/min on a BEH C18 column.

The selection of the analytical column plays a crucial role in the development of LC-MS methods for the simultaneous determination of multiple mycotoxins [27]. Accordingly, four reversed-phase columns differing in stationary phase and length (Acquity UPLC HSS T3, Acquity UPLC BEH C18, Zorbax RRHD Eclipse Plus C18 and Zorbax Eclipse Plus C8; details in Section 4.4) were compared for the simultaneous separation of the target analytes. The C8 and BEH C18 columns gave broad or missing TeA peaks and poor retention of the more polar compounds, while the HSS T3 column provided the best signal intensity, retention and peak shape for all analytes (Figures S1 and S2), with a higher PAT signal intensity. This behaviour is consistent with the trifunctionally bonded high-strength-silica C18 chemistry of the HSS T3 column, which enhances the retention of polar analytes over a wide pH range [40]. In all cases, however, a partial coelution of TEN and AOH was observed. Therefore, other chromatographic parameters affecting the separation, such as column temperatures (20, 25, 30, and 40 °C) and flow rate (0.3, 0.35, and 0.4 mL/min), were further investigated. The best chromatographic performance was achieved at a column temperature of 25 °C and a flow rate of 0.4 mL/min. Consequently, the HSS T3 column, operated under these optimized conditions, was selected for the subsequent analyses.

### 2.3. Optimization of the Sample Preparation

In this study, different extraction and purification procedures, such as solid-liquid extraction (SLE), modified salting-out-liquid-liquid extraction (SALLE), and UAE, as well as SPE and d-SPE, were tested and compared based on recovery experiments for each target analyte.

Acetonitrile was selected as an extraction solvent because of its ability to extract analytes of different polarity while limiting the co-extraction of interferents [15,38]. The effect of acidification of this organic phase was evaluated by adding 1% formic acid or 1% acetic acid. Both acidified solvents improved recoveries compared with non-acidified acetonitrile, with 1% formic acid exhibiting the highest initial recoveries (above 65% for all analytes), in agreement with previous reports [41,42]. The addition of NaCl, commonly employed as a salting-out agent to promote the partitioning of analytes into the organic phase [15,42], provided no improvement in extraction efficiency in the fruit purée matrix. Slightly lower recoveries were obtained in most cases, likely due to altered analyte solubility or phase distribution in the sugar-rich purée. Consequently, NaCl was omitted from the extraction procedure in order to reduce reagent consumption and simplify the sample preparation workflow.

Different agitation modes were first evaluated by comparing mechanical shaking with UAE at an extraction time of 10 min. UAE provided recoveries approximately 10% higher for PAT and AOH and it was therefore selected for further optimization. Subsequently, the effect of sonication time (e.g., 5, 10, and 20 min) was investigated. A sonication time of 5 min was insufficient, whereas no significant improvement was observed when increasing the extraction time from 10 to 20 min. Therefore, UAE for 10 min was selected as the best compromise between extraction efficiency (recoveries 80–90%) and analysis time, in accordance with the principles of green sample preparation [35]. Finally, different dispersive sorbents (C18, PSA, Z-Sep+ and Florisil) were compared with a conventional Oasis HLB SPE clean-up, included as a reference method since it has previously provided the best purification performance for the determination of PAT and *Alternaria* toxins [31,38], in terms of recovery (Figure 1a) and matrix effect (Figure 1b). At an earlier preliminary stage, TeA had also been evaluated but showed poor and highly variable recoveries across all sorbents (1.6–149.3%) and was therefore excluded from further optimization. SPE yielded poor purification performance for all analytes, probably because of strong analyte retention, which may have resulted in a partial analyte elution. Among the dispersive sorbents, Florisil provided the best balance of high recovery and low matrix effect; further optimization of the Florisil amount (50–200 mg) revealed a trade-off between recovery and matrix effect, with the best recovery values for each analyte at 50 mg, while increasing the Florisil amount improved the matrix effect at the cost of recovery. Since recovery was prioritized and the residual matrix effect was adjusted through the procedural calibration curve, 50 mg was selected as the final amount, also minimizing reagent consumption. Based on these findings, the final optimized procedure combining UAE with a d-SPE clean-up using 50 mg of Florisil was adopted (see Figure S3, Supplementary Materials).

### 2.4. Method Validation

The optimized UAE-d-SPE-UHPLC-MS/MS method was validated in terms of linearity, limits of detection (LODs), limits of quantification (LOQs), precision, recovery rates (%R) and matrix effect (%ME). A blank fruit-mix purée was selected as the representative matrix, as no targeted compounds were detected above the LOD. Linearity was evaluated with a procedural calibration curve, obtained by spiking blank samples at different concentration levels (0.1; 0.5; 1; 2.5; 5; 10; 25; 50; 100 µg/kg) before performing the whole extraction procedure, so that matrix effects and analyte losses were corrected within the calibration. The calibration curves were fitted by least-squares regression and evaluated through the determination coefficient (R^2^). The LOQ was determined as the lowest level of the matrix-matched calibration curve corresponding to each analyte giving a signal-to-noise ratio of 10, and the LOD was calculated as 3/10 of the LOQ. Good linearity was observed, with determination coefficients (R^2^) higher than 0.98. The LOQs were 0.1 µg/kg for AOH, AME and TEN and 0.5 µg/kg for PAT, well below the maximum level of 10 µg/kg set for PAT in food for infants and young children by Regulation (EU) 2023/915, confirming the adequate sensitivity of the method for the intended application. Precision was assessed at three concentration levels (5, 10 and 25 µg/kg) and expressed as the relative standard deviation (RSD) calculated from the resulting concentrations obtained for the replicate analyses. Precision was evaluated both as intra-day repeatability (three injections on the same day) and as inter-day precision (with the procedure repeated over three consecutive days), with all RSD values meeting the acceptance limit of 20%. Recovery was assessed at the same three concentration levels (5, 10 and 25 µg/kg) as the ratio between samples fortified before and after the extraction step. The samples were extracted in duplicate and injected in triplicate (*n* = 6). Recovery ranged from 76 to 112%. These levels were selected as validation levels to assess method performance below, at, and above the regulated concentration, respectively, ensuring reliable quantification of PAT at and around the regulatory level.

The matrix effect was calculated from the slopes of the procedural and solvent calibration curves. Although all analytes showed signal suppression, especially for PAT (Table S2), the use of procedural calibration curves effectively compensated for these effects, minimizing potential quantification errors in real samples.

Overall, all validation parameters were evaluated following recommendations of SANTE/11312/2021, and the Commission Implementing Regulation (EU) 2023/2782 [43] on 1 April 2024, which has since introduced mycotoxin-specific performance criteria, including a maximum LOQ ≤ 0.5 × ML for regulated analytes. The analytical performance of the method was therefore also assessed in relation to the applicable requirements of this Regulation. This confirmed the suitability of the method for the accurate quantification of PAT and *Alternaria* toxins in commercial infant fruit purées. Detailed linearity and LOD/LOQ values, precision (RSD%) data, and recovery (%R) data are reported in Tables S2–S4, respectively.

### 2.5. Occurrence in Commercial Samples

The validated method was applied to forty commercial fruit-mix purées with varying apple content collected from the Italian (*n* = 20) and Spanish (*n* = 20) markets, whose composition, apple content and organic/conventional status are reported in Tables S5 and S6. PAT and the *Alternaria* toxins AOH and AME were widely quantified and frequently co-occurred within the same sample, whereas TEN was never detected (Figure 2). Differences between the two countries were assessed with the non-parametric Wilcoxon rank-sum test (AOH: W = 58, *p* = 0.126; AME: W = 99, *p* = 0.423), justified by the small group sizes and the non-normal, skewed distribution of the data. PAT was not statistically compared between countries due to the limited number of PAT-positive samples above the LOQ (*n* = 5 in Italy, *n* = 2 in Spain). The Italian samples showed a higher frequency for all three toxins, whereas concentration patterns varied by toxin, with AOH and AME tending to be higher in Spain. PAT, AOH and AME occurred in 25%, 75% and 80% of the Italian samples and in 10%, 60% and 75% of the Spanish samples, respectively. The same occurrence pattern (PAT < AOH < AME) was observed in both countries (Figure 2b).

PAT concentrations ranged from 1.16 to 11.60 µg/kg in the Italian samples and from 1.48 to 2.81 µg/kg in the Spanish samples; one Italian sample (11.60 µg/kg) exceeded the maximum level of 10 µg/kg established by Regulation (EU) 2023/915 [21]. AOH and AME reached 8.67 and 4.60 µg/kg in the Italian samples, respectively, and 6.92 and 5.90 µg/kg in the Spanish samples, respectively (Figure 2c). The widespread co-occurrence of AOH and AME in both markets aligns with the climate-driven scenario proposed for Mediterranean regions, where rising temperatures are expected to promote the occurrence of both *Alternaria* toxins [23,24,44]. The complete concentration values and co-occurrence results are reported in Tables S7 and S8 (Supplementary Materials).

Although the estimated LOQ of 0.5 µg/kg was sufficient to establish the presence of PAT based on the applied identification criteria, recovery and precision were not validated at concentrations below 5 µg/kg. Therefore, PAT concentrations close to the estimated LOQ should be interpreted with caution. Nevertheless, concentrations above 5 µg/kg were within the experimentally validated range and could therefore be reliably quantified for the purpose of assessing compliance with the applicable regulatory requirements.

The possible relationship between apple content and mycotoxin concentration was explored by Spearman’s rank correlation (Figure 3). As noted above, PAT was excluded from this analysis due to the low number of positive samples, particularly in the Spanish market (*n* = 2; Figure 2a), which precluded a meaningful correlation analysis. Additionally, one sample per market lacking a declared apple percentage (ITA_17 and ESP_16) was excluded from the apple content–mycotoxin correlation analysis. Correlation analysis was restricted to samples with quantifiable concentrations (AOH: *n* = 14 and 11; AME: *n* = 15 and 14, in Italy and Spain, respectively); as such, these correlations characterize the relationship between apple content and toxin levels among quantified samples only and do not capture patterns related to non-detected samples. No significant association was found between apple content and the concentration of either *Alternaria* toxin. The strongest association was observed for AOH in the Spanish samples (ρ = 0.579, *p* = 0.062), while it was less pronounced in the Italian samples (ρ = 0.396, *p* = 0.161). Apples are known to be highly susceptible to fungal colonization at all production stages, particularly by *Alternaria* species responsible for mouldy core rot [9], although the present sample size did not provide sufficient statistical power to confirm this relationship in either market. Correlations for AME were weak and non-significant in both countries (ITA: ρ = 0.132, *p* = 0.64; ESP: ρ = 0.293, *p* = 0.309). The generally weak and inconsistent correlations, together with the scattered distribution of the data, are most likely due to the limited number of samples and to the contribution of the other fruits present in the purées, indicating that more extensive sampling is needed to fully clarify the relationship between apple content and mycotoxin occurrence.

### 2.6. Dietary Exposure, Risk Characterization, and Exposure Patterns

The selected consumer-only mean consumption values are shown in Table S9. The occurrence dataset comprised 20 samples from each country for AME, AOH, PAT and TEN. Lower-bound (LB) and upper-bound (UB) means are summarized in Table S10. AOH concentrations were similar between countries, whereas the AME mean was higher in Spain and the PAT mean was higher in Italy. TEN was not reported numerically in any sample and therefore contributed only under the UB substitution scenario. The LB–UB difference was minimal for AOH and AME, reflecting the predominance of reported numeric values. In contrast, uncertainty from left censoring was proportionally larger for PAT and TEN.

Across both countries, age groups and sex strata, the EDI ranged from 0.00710 to 0.02449 µg/kg bw/day for AOH, 0.00364 to 0.01786 µg/kg bw/day for AME, 0.00148 to 0.0086 µg/kg bw/day for PAT and 0 to 0.000382 µg/kg bw/day for TEN. These ranges extend from the lowest LB estimate to the highest UB estimate. Country-, age- and sex-specific LB–UB estimates are displayed in Figure 4A. The complete numerical EDI estimates and corresponding risk-characterization ratios for each country, age group, sex and scenario are provided in Supplementary Table S11.

For Italy, AOH exposure ranged from 0.00710 to 0.01289 µg/kg bw/day, followed by PAT (0.00394–0.0086), AME (0.00364–0.00663) and TEN (0–0.000202 µg/kg bw/day). The highest Italian UB estimates occurred in female toddlers. For Spain, AOH exposure ranged from 0.01323 to 0.02449 µg/kg bw/day, followed by AME (0.00966–0.01786), PAT (0.00148–0.00445) and TEN (0–0.000382 µg/kg bw/day). Spanish female infants had the highest UB exposure to all toxins. Spain had higher UB exposure to AOH, AME and TEN in every matched age–sex stratum. Conversely, PAT exposure was consistently higher in Italy, with Spain-to-Italy UB ratios ranging from 0.31 to 0.81. Because exposure was calculated from country-specific mean concentrations and mean consumption, these contrasts reflect both occurrence and consumption. The direction and magnitude of each matched country contrast are shown in Figure 4B.

AOH and AME exceeded their respective threshold of toxicological concern (TTC) screening values in every age–sex stratum under both LB and UB assumptions. Across all strata, the AOH exposure to TTC ratios ranged from 2.84 to 9.79 and those for AME ranged from 1.46 to 7.14. The highest ratios were observed for Spanish female infants. These findings indicate screening-level exposure concern according to the TTC-based framework and should not be interpreted as quantitative evidence of adverse health effects.

In contrast, the highest PAT HQ was 0.022 (2.2% of the provisional maximum tolerable daily intake (PMTDI)), while the maximum TEN exposure-to-TTC ratio was 0.000255 (0.025% of the TTC). The UB estimates are summarized by toxin and age group in Figure 5A and across all population strata in Figure 5B.

Expressed as a percentage of the reference value or screening value in the UB scenario, AOH reached 285–979% and AME 147–714% of the TTC. The corresponding maximum values were 2.22% of the PMTDI for PAT and 0.025% of the TTC for TEN.

Under the UB scenario, the cumulative exposure to AOH and AME, expressed as a hazard index (HI), ranged from 4.30 to 7.81 in Italy and from 9.21 to 16.94 in Spain, with the highest value observed in Spanish female infants. In this context, the HI represents a screening-level indicator of combined exposure and should be interpreted with caution not as a definitive quantitative estimate of combined toxicity.

In Italy, female estimates exceeded male estimates by 55.3% in infants, 12.4% in toddlers, and 77.9% in other children. In Spain, female estimates were 13.0% higher in infants, whereas male estimates were 9.3% higher in toddlers and 43.8% higher in other children. Consequently, the highest UB estimates for all four toxins occurred in female toddlers in Italy and in female infants in Spain. The within-country female–male contrasts are shown in Figure 6A.

### 2.7. Comparative Discussion on Occurrence, Dietary Exposure and Risk Characterization

AOH and AME exceeded the safety threshold values in every investigated population stratum, whereas PAT and TEN remained well below their respective reference values. This pattern is consistent with the European dietary assessment, in which AOH and AME exposure exceeded the 2.5 ng/kg bw/day TTC in several population groups [45]. An important consideration in interpreting the present findings is the toxicological uncertainty underlying the TTC approach. AOH and AME have shown genotoxic effects in vitro and EFSA applied the 2.5 ng/kg bw/day TTC as a conservative screening framework, and concluded that exposure estimates above this value indicated a need for additional compound-specific toxicological information. Accordingly, the TTC exceedances observed in the present study should not be interpreted as equivalent to exceedance of a health-based guidance value or as quantitative evidence of adverse health effects [46].

The findings are similar to those of the recent Italian survey of 62 commercial apple purées by Carbonell-Rozas et al. [17]. That study reported PAT in 92% of samples and widespread *Alternaria* toxin occurrence, with AOH exposure reaching approximately 0.025 µg/kg bw/day and combined AOH + AME screening above the safety threshold, while PAT exposure was negligible. The maximum AOH EDI in the present assessment (0.02449 µg/kg bw/day in Spanish female infants) was nearly identical to that reported in the previous Italian study [17]. In contrast, the maximum Italian PAT EDI in the present analysis (0.0086 µg/kg bw/day) was around one order of magnitude lower than the 0.075–0.096 µg/kg bw/day reported in the Italian survey, consistent with the lower mean PAT occurrence used here 1.15–1.28 µg/kg versus 12.5 µg/kg among positive samples in the published survey [17].

The prioritization of AOH and AME is further supported by the Argentinean assessment of apple by-products [47]. In apple-based infant food, mean concentrations among positive samples were 5.0 µg/kg for AOH, 6.5 µg/kg for AME, and 30.7 µg/kg for TEN. Their analysis estimated TTC exceedance for AME in 84.9–91.3% of children and for AOH in 6.3–47.2% of children, depending on age and censoring scenario [47].

The low PAT HQ values should not be interpreted as a substitute for lot-by-lot compliance control. Mean-occurrence exposure assessment and regulatory compliance address different questions. The highest Italian result was 11.60 µg/kg PAT and therefore slightly exceeded the 10 µg/kg maximum level for fruit-based foods intended for infants and young children under Regulation (EU) 2023/915 [21], whereas the highest chronic HQ based on mean occurrence was only 0.022. A similar contrast was reported in Belgium, where PAT occurred in 3 of 42 purées, the overall mean was 2.4 µg/kg, and one sample reached 35.9 µg/kg and exceeded the infant/young-child limit [48]. Accordingly, the mean chronic exposure result supports a low average PAT intake relative to the PMTDI, while the proximity of the Italian maximum to the regulatory limit supports continued batch surveillance.

Monitoring the final processed product is especially important because thermal treatment can eliminate viable fungi without necessarily eliminating toxins. In an industrial apple-purée line, Carbonell-Rozas et al. [49] detected PAT and *Alternaria* toxins before and after processing and found no significant reduction attributable to the 95–105 °C treatment; PAT was significantly higher in the final purée on a dry-matter basis, potentially owing to increased extractability and matrix changes [49]. Broader reviews likewise show substantial heterogeneity in PAT occurrence across fruit purées and compotes in Italy, Spain and other countries [6,50]. These data support preventive control of raw-fruit quality together with surveillance of finished purées.

The higher Spanish exposure to AOH and AME resulted from the combination of greater AME occurrence and generally greater consumption of the selected Spanish food category. Meanwhile, PAT occurrence was higher in Italy, producing higher PAT exposure in every matched age–sex group despite the larger Spanish consumption values in several strata. Our findings indicate that Spain was not uniformly the higher-exposure country and that the direction of the difference depended on the toxin and its occurrence profile.

Infants and toddlers were often among the highest-exposure groups due to their relatively high food intake per kilogram of body weight. This observation is consistent with the broader infant-food literature, which identifies low body weight and immature detoxification capacity as important determinants of vulnerability [6,50,51,52,53].

Key strengths of this study are the use of country-specific occurrence data, sex-specific consumption among consumers, separate infant, toddler and other-child strata, and explicit LB and UB treatment of left-censored results. The small LB–UB separation for AOH and AME indicates that their safety threshold exceedance is not driven primarily by non-detect substitution. The use of final-product occurrence data also aligns with evidence that processing does not reliably remove preformed apple mycotoxins [49].

The study presents several uncertainties. First, the occurrence data were based on 20 samples per country and may not capture seasonal, regional, or batch variability. Second, the primary exposure assessment was deterministic and based on country-specific mean concentrations and consumer-only mean consumption. Consequently, it provides a central exposure estimate and does not characterize the full distribution of individual exposure, high consumers, or potential concentration–consumption correlations.

Third, the assessment presented herein covers the quantitative exposure and risk associated with PAT, AOH, AME and TEN; however, it does not represent a complete multiclass assessment considering that other mycotoxins have been previously reported in apple fruit purées.

### 2.8. Suspect Screening Analysis via UHPLC-Orbitrap-MS

The complete Mass List is provided as Supplementary Table S12. Tentative identifications were further supported using the Fragment Ion Search (FISh) algorithm implemented in Compound Discoverer, which evaluates the agreement between the experimental and predicted fragmentation patterns.

After applying the workflow, the number of detected features was reduced from 4011 to 190. These candidates were then manually reviewed based on the matching scores obtained from the different spectral libraries, with particular emphasis on the Mass List. The target mycotoxins PAT, AME, AOH, and TEN were successfully identified in the QC samples, confirming the reliability of the workflow. In addition, TeA was identified in 5 out of 40 samples and confirmed using an analytical standard. This finding highlights the value of including TeA in future regulatory frameworks. Furthermore, it supports our preliminary selection of the chromatographic column, which also proved capable of detecting this compound, although the method was not validated for its quantitative determination.

Furthermore, altenusin (ALT) was putatively identified with a confidence Level 2 based on the Schymanski classification, indicating a probable structure substantiated by diagnostic MS/MS fragments and spectral library similarity [54]. Aflatoxin G2 (AFG2) was assigned confidence Level 3 according to the Schymanski classification, as the molecular formula could be determined from the accurate mass data; however, no MS/MS fragmentation data were available to support structural identification.

Beyond these new findings, the high-resolution suspect screening workflow facilitated the annotation of the previously unidentified peak that was consistently detected in the fruit-mix purée samples, eluting in close proximity to PAT. The compound was identified as 2,3-dihydroxybenzoic acid (RT = 2.8 min). Previous studies have demonstrated that this compound may be misidentified as PAT during HRMS library searches because of its identical elemental composition and similar fragmentation pattern, highlighting the importance of adequate chromatographic separation and careful spectral interpretation [55]. Although the chromatographic conditions employed in this study provided adequate separation of both compounds, identifying this potential interferent is essential, as its similar fragmentation pattern could result in misidentification and false-positive detections in targeted approaches, compromising reliable quantification and regulatory compliance. 2,3-dihydroxybenzoic acid is a naturally occurring compound with documented biosynthetic origins in both plants, as a salicylic acid-derived metabolite induced during the plant response to fungal infection [56], and also caused by fungal metabolic activity [57]. In this context, its presence in this matrix could be plausible due to either host-plant defense responses or fungal metabolic activity linked to the production of both PAT and *Alternaria* toxins.

## 3. Conclusions

A rapid and fit-for-purpose UAE-d-SPE-UHPLC-MS/MS method was successfully developed and validated for the simultaneous determination of PAT and the *Alternaria* toxins in commercial fruit purées intended for infant consumption. The method provided satisfactory sensitivity, accuracy, and precision and proved suitable for routine monitoring of these contaminants.

Targeted analysis revealed the frequent co-occurrence of PAT, AOH, and AME in fruit purées from both the Italian and Spanish markets. Although PAT exposure remained well below its health-based guidance value, AOH and AME exceeded their respective TTC values in all evaluated population groups, identifying these toxins as the main contributors to dietary risk. These findings highlight the need for continued monitoring and further toxicological assessment of *Alternaria* toxins in foods intended for infants and young children.

In addition, the HRMS suspect-screening workflow expanded the mycotoxin profile beyond the targeted analytes, enabling the tentative identification of TeA, ALT, and AFG2, as well as the annotation of 2,3-dihydroxybenzoic acid, a compound that may interfere with PAT identification during HRMS library searches or targeted methods when chromatographic separation is insufficient. These results demonstrate the complementary value of HRMS suspect screening for improving contaminant characterization and avoiding false-positive identifications in complex fruit-based matrices.

Overall, this study provides an integrated analytical and risk assessment framework combining targeted UHPLC-MS/MS quantification, HRMS suspect screening, and dietary exposure assessment, contributing valuable occurrence data for infant fruit purées and supporting future monitoring strategies and regulatory decision-making regarding emerging mycotoxins.

## 4. Materials and Methods

### 4.1. Chemicals and Reagents

Analytical standards of PAT were purchased from Fermentek Ltd. (Jerusalem, Israel) and the *Alternaria* toxins AOH, AME, TEN and TeA from Biopure (Getzersdorf, Austria). Ultrapure water and methanol were obtained from PanReac AppliChem (Gatersleben, Germany), acetonitrile and sodium chloride from Honeywell (Charlotte, NC, USA) and formic acid from Fisher Scientific (Erembodegem, Belgium). Acetic acid, ammonium acetate and DSC-18 SPE material were from Sigma-Aldrich (Darmstadt, Germany), Oasis HLB cartridges from Waters (Milford, MA, USA), the dispersive sorbents Z-Sep+ and PSA from Supelco (Darmstadt, Germany) and Florisil from J.T. Baker (Deventer, The Netherlands).

### 4.2. Instrumentation

Target quantification was performed on an Agilent 1290 Infinity II LC system coupled to an Agilent 6460 triple-quadrupole mass spectrometer fitted with an electrospray ionization source (Agilent Technologies, Santa Clara, CA, USA). Data acquisition and quantitative analysis were carried out with Agilent MassHunter software (version 10.1, Agilent Technologies, Santa Clara, CA, USA). Suspect screening was performed on a Vanquish LC chromatograph (Thermo Fisher Scientific, Waltham, MA, USA), comprising a binary pump, a degasser, a temperature-controlled column compartment and an autosampler, coupled to a Q-Exactive hybrid quadrupole-Orbitrap high-resolution mass spectrometer (Thermo Fisher Scientific, Waltham, MA, USA). UHPLC-Q-Orbitrap-HRMS data were acquired using Xcalibur Sequence Setup software (Version 4.4, Thermo Fisher Scientific, Waltham, MA, USA). Additional equipment used for sample treatment included a Pioneer analytical balance and a Frontier 5816 centrifuge (Ohaus, Nänikon, Switzerland), a ZX4 vortex mixer (Velp Scientifica, Usmate Velate, Italy), an Elmasonic S 80H ultrasonic bath (Elma Schmidbauer, Singen, Germany), a Reax 2 rotatory agitator (Heidolph Instruments, Schwabach, Germany) and an SBH130D/3 block heater (Stuart, Cole-Parmer, Stone, UK).

### 4.3. Sample Collection and Sample Treatment

Forty fruit-mix purées for infant consumption, with varying apple content and labelled as organic or conventional, were randomly purchased from retail markets in Italy (*n* = 20) and Spain (*n* = 20) between February and March 2024. Sampling was designed to characterize dietary exposure at the point of consumption in each country, and products were therefore included regardless of their country of manufacture or raw material origin. Where the same brand or product line was available in both markets, the retail units purchased independently in Italy and Spain were treated as separate market samples within the respective national datasets. Several units of each product were combined and homogenized to obtain representative samples, which were stored at 4 °C until analysis.

Each sample was analyzed in duplicate (two independent extractions, A and B), and each extract was injected twice into the UHPLC-MS/MS system. For each extraction, the peak areas of the two injections were averaged, and the resulting mean area was converted to concentration using the analyte-specific procedural calibration curve. The concentration obtained from the two independent extractions was then used to calculate the mean ± SD (*n* = 4).

A blank fruit-mix purée free of detectable target analytes was used for method development and for the procedural calibration curve. The composition and apple content of the samples are reported in Tables S5 and S6 (Supplementary Materials). The proposed sample treatment was as follows.

One gram of homogenized purée was weighed into a 15 mL tube and extracted with 5 mL of acetonitrile containing 1% formic acid. The mixture was vortexed for 10 s, sonicated at 30 °C for 10 min and centrifuged at 7500 rpm (8170× *g*) for 10 min at 4 °C. A 2.5 mL aliquot of the supernatant was cleaned up with 50 mg of Florisil by vortexing for 1 min and centrifuging under the same conditions. A 2.5 mL portion of the purified extract was evaporated to dryness under a nitrogen stream at 40 °C, reconstituted in 0.5 mL of acetonitrile/water (5:95, *v*/*v*), vortexed for 1 min and filtered through a 0.2 µm PTFE syringe filter (13 mm; Agilent Technologies, Santa Clara, CA, USA) prior to injection.

### 4.4. UHPLC-MS/MS Conditions

Column selection was optimized by comparing four candidate columns: an Acquity UPLC HSS T3 column (2.1 × 100 mm, 1.8 µm, 100 Å; Waters, Milford, MA, USA), an Acquity UPLC BEH C18 column (2.1 × 50 mm, 1.7 µm, 130 Å; Waters, Milford, MA, USA), a Zorbax RRHD Eclipse Plus C18 column (2.1 × 50 mm, 1.8 µm, 95 Å; Agilent Technologies, Santa Clara, CA, USA) and a Zorbax Eclipse Plus C8 column (2.1 × 100 mm, 1.8 µm, 95 Å; Agilent Technologies, Santa Clara, CA, USA). The HSS T3 column provided the best chromatographic performance for the target analytes and was therefore selected for the final method. Chromatographic separation was performed on an Acquity UPLC HSS T3 column at 25 °C, with an injection volume of 5 µL and a flow rate of 0.4 mL/min. Mobile phase A was ultrapure water with 0.05% acetic acid and 5 mM ammonium acetate, and mobile phase B was acetonitrile with 0.05% acetic acid. The gradient was 5–30% B (0–2 min), 30–100% B (2–8 min) and 100% B (8–10 min), followed by re-equilibration, for a total run time of 12 min; the elution order was PAT, TeA, TEN, AOH, and AME. The mass spectrometer was operated in MRM mode in both polarities (PAT, AOH, AME and TeA in negative mode; TEN in positive mode), with a nebulizer pressure of 45 psi, capillary voltage 3500 V, gas temperature 350 °C, sheath-gas temperature 300 °C, sheath-gas flow 11 L/min and drying-gas flow 5 L/min. The monitored transitions and compound-specific parameters are listed in Table S1.

### 4.5. Qualitative Analysis of the Real Samples

Data were processed using R version 4.4.1 (R Core Team, R Foundation for Statistical Computing, Vienna, Austria; packages ggplot2, reshape2 and patchwork for data handling and visualisation). Normality was assessed by the Shapiro–Wilk test, given the departure from normality observed for most groups, the small group sizes and the left-censored nature of concentration data, differences between Italian and Spanish samples were evaluated using the two-sided Wilcoxon rank-sum (Mann–Whitney U) test, restricted to positives samples (concentrations above LOQ). The relationship between apple content and mycotoxin concentration was assessed by Spearman’s rank correlation coefficient (ρ), computed on positive samples with a declared apple percentage. Statistical significance was set at *p* < 0.05. Given the exploratory and descriptive nature of these comparisons and small sample sizes, no correction for multiple comparisons (e.g., Bonferroni) was applied; therefore, results should be interpreted as preliminary rather than confirmatory.

### 4.6. Food Consumption and Occurrence Data

Consumer-only mean intakes expressed as g/kg body weight (bw)/day were extracted separately for infants, toddlers and other children and by sex from the IV SCAI CHILD (2017–2020) [58] and ENALIA (2013–2014) [59] surveys. For both national surveys, the FoodEx2 category “Fruit or fruit–vegetable purees” was used as the closest available consumption category for the commercial fruit-mix purées investigated in the present study. Because a more specific category corresponding to the exact formulation and variable apple content of the sampled products was not available, no additional adjustment was applied. The food consumption data is presented in the Supplementary Material (Table S9).

Country-specific occurrence data comprised 20 fruit-mix purée samples from Italy and 20 from Spain, with varying apple content. Quantitative assessment was performed for AME, AOH, PAT and TEN, with concentrations expressed as µg/kg. For lower-bound (LB) estimates, non-detects and results reported as <LOQ were assigned a value of zero. For upper-bound (UB) estimates, non-detects were assigned the applicable LOD, while results >LOD but <LOQ were assigned the applicable LOQ. Explicit numeric results, including reported values below the nominal LOQ, were retained. Arithmetic country- and toxin-specific LB and UB mean concentrations were then calculated.

### 4.7. Deterministic Dietary Exposure Assessment and Risk Characterization

Chronic estimated daily intake (EDI) was calculated independently for each country, age group, sex, toxin and censoring scenario by combining the country-specific mean concentration with the corresponding consumer-only mean intake. The deterministic calculation was:

EDI (µg/kg bw/day) = [mean concentration (µg/kg) × consumption (g/kg bw/day)]/1000.

LB and UB EDIs were generated using the corresponding LB and UB occurrence means.

For PAT, the hazard quotient (HQ) was calculated as the EDI divided by the provisional PMTDI of 0.4 µg/kg bw/day [60]. For AOH and AME, the EDI was divided by the TTC of 0.0025 µg/kg bw/day, and for TEN by the TTC of 1.5 µg/kg bw/day [45]. The resulting ratios for AOH, AME and TEN were interpreted as exposure-to-TTC ratios within a screening-level framework. Because AOH and AME are genotoxic *Alternaria* toxins evaluated within the same TTC-based framework, the combined assessment was performed assuming dose additivity. The resulting HI was considered a lower-tier, screening-level indicator of cumulative exposure. Ratios were also expressed as percentages of the corresponding reference value; values above 1, or 100%, were considered screening-level exceedances. For the cumulative exposure to AOH + AME we used the hazard index (HI).

HQ*_i_* = EDI*_i_*/HBGV*_i_*

where HQ = hazard quotient, EDI = estimated daily intake, and HBGV = health-based guidance value.

HI = ΣHQ*_i_* = Σ(EDI*_i_*/HBGV*_i_*)

where HI = hazard index, and ΣHQ = sum of hazard quotients.

### 4.8. UHPLC-Orbitrap-HRMS Conditions

Chromatographic separation was performed using the same column and mobile phases indicated in Section 4.4.

The HRMS operated in both positive and negative ESI modes, using the following parameters: the heater temperature was 300 °C and the capillary temperature was 300 °C. The auxiliary and sheath gas used was nitrogen (95%), the spray voltage was 4 kV, and the S-lens radio frequency level was 50 (arbitrary units). Full-scan mode was used for HRMS data acquisition of precursor ions in the mass-to-charge ratio (*m*/*z*) range of 50–750 for both ESI+ and ESI– modes, with a maximum injection time (IT) of 250 ms, an automatic gain control (AGC) target of 1 × 105, and a resolution of 70,000 fwhm (*m*/*z* 200). For the monitoring of fragment ions, data-dependent acquisition (DDA) was performed at a collision energy of 30 eV and a maximum injection time of 125 ms.

A comprehensive workflow for the suspect screening analysis of known mycotoxins in fruit purées samples was developed using Compound Discoverer version 3.3 software (Thermo Fisher Scientific, Thermo Fisher Scientific, Waltham, MA, USA).

Initially, raw data (.raw files) were imported into the software. Calibration standards, procedure blanks, and quality control (QC) samples were classified as “standard”, “blank”, and “quality control”, respectively. Background filtering was performed using a maximum sample-to-blank ratio of 5, and QC samples were used for area refinement.

The selected settings for Compound Discoverer were as follows: mass tolerance of 5 ppm, minimum peak intensity of 100,000, intensity tolerance of 30%, signal-to-noise (S/N) threshold of 3, intensity threshold of 0.1%, and retention time (RT) tolerance of 0.5 min. The preferred adducts included [M + H]+, [M + H − H2O]+, [M + Na]+, [M − H]−, and [M + K]+.

Compound identification was performed by matching against LC-MS spectral libraries, including ChemSpider, ChEBI, ChemBank, DrugBank, EPA Toxcast, FDA, MassBank, NIST, PubMed, Toxin (Toxin-Target Database), LIPID maps, PlantCyc, PubMed, Natural Products Atlas 2021_8, mzCloud, and mzVault, with a mass tolerance of 5 ppm for precise annotations. In addition, a Mass List containing the exact masses of mycotoxins, their metabolites, and compounds belonging to the major mycotoxin families that could be potentially found in fruits (e.g., aflatoxins, trichothecenes, fumonisins, zearalenones, ochratoxins) was incorporated into the workflow and used to filter features during the suspect screening analysis. To ensure reliable annotations, only features meeting the criteria of ±5 ppm mass tolerance, peak rating ≥ 4, and RT between 1 and 15 min were considered.

## Figures and Tables

**Figure 1:**
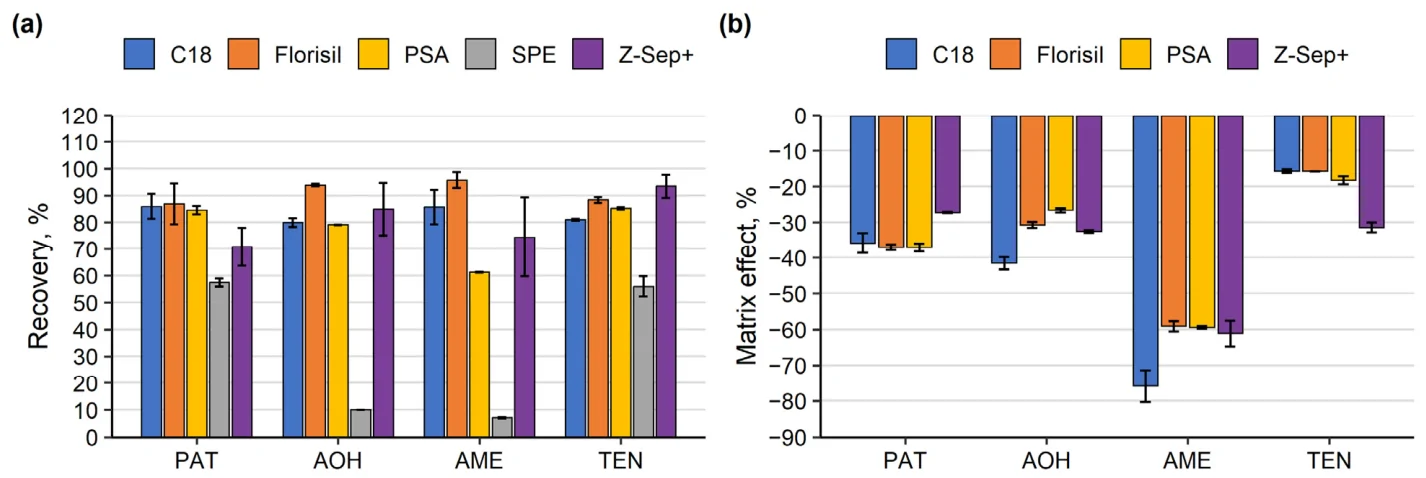
(**a**) Recovery (%) and (**b**) matrix effect (%) for the target mycotoxins (PAT, AOH, AME, TEN) obtained using different clean-up approaches: dispersive sorbents (C18, Florisil, PSA and Z-Sep+) and Oasis HLB SPE. Data are expressed as mean ± SD. Error bars represent standard deviation of individual replicates (*n* = 4 for recovery; *n* = 2 for matrix effect). SPE was excluded from the matrix-effect evaluation (**b**) due to its poor recovery performance.

**Figure 2:**
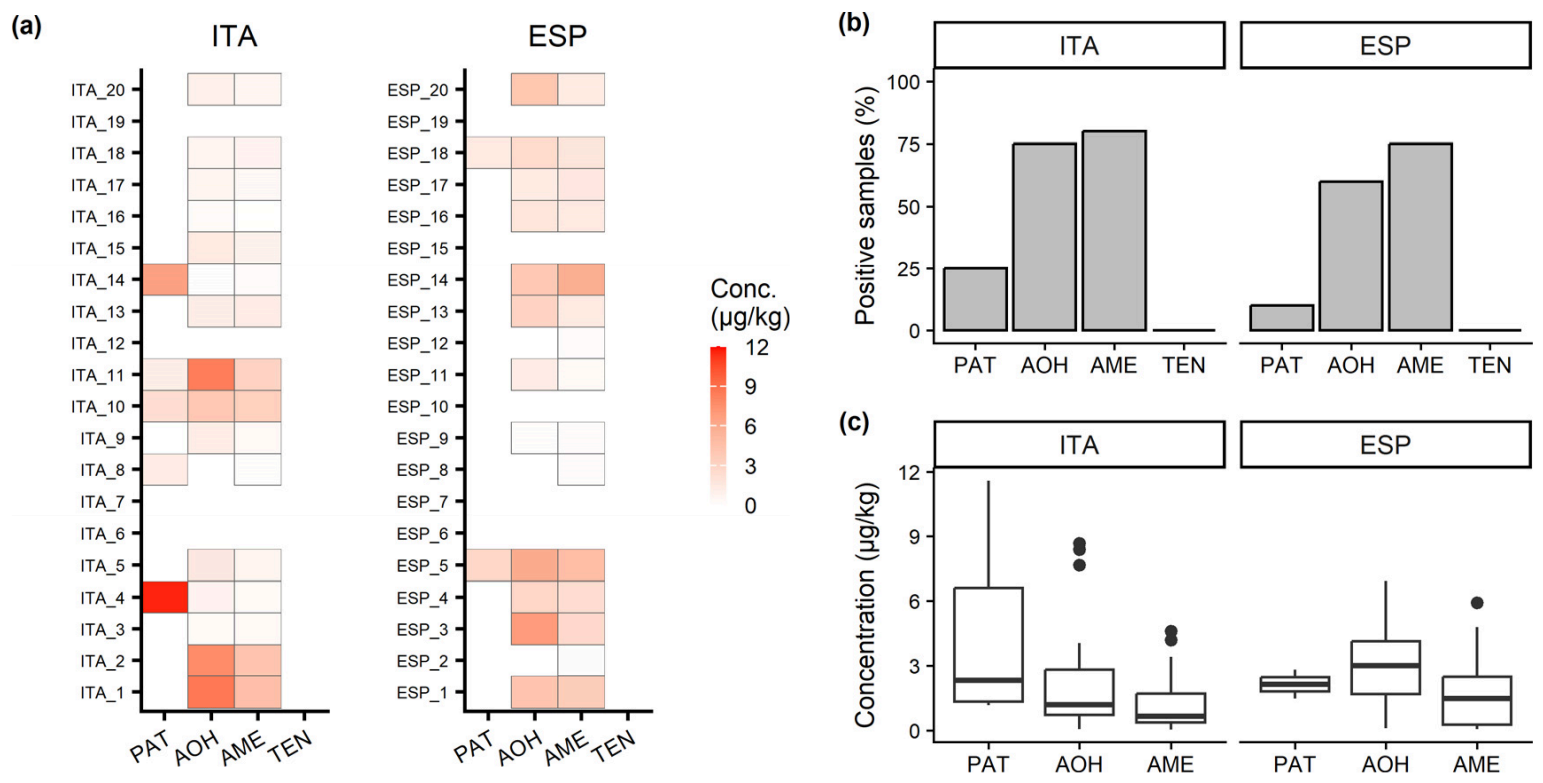
(**a**) Occurrence and concentration (µg/kg) of PAT and *Alternaria* toxins (AOH, AME, TEN) in the Italian (ITA, *n* = 20) and Spanish (ESP, *n* = 20) commercial fruit-mix purées; only values ≥ LOQ are shown; (**b**) number of positive samples (%); and (**c**) concentration distributions (µg/kg) of patulin and *Alternaria* toxins in the Italian and Spanish fruit-mix purées. Dots in (**c**) represent outlier values beyond 1.5 times the interquartile range.

**Figure 3:**
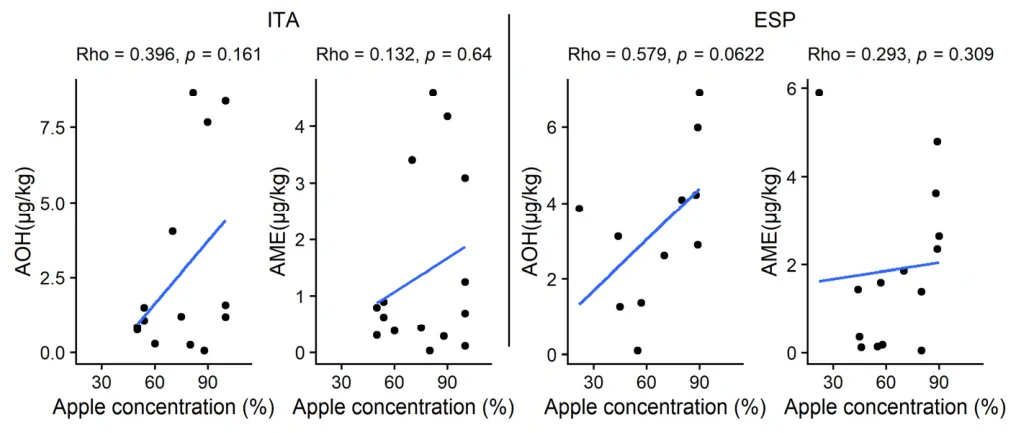
Spearman’s rank correlation between apple content (%, x-axis) and mycotoxin concentration (µg/kg, y-axis) in the Italian and Spanish fruit-mix purées. Dots represent individual sample values; blue lines indicate the linear regression fit.

**Figure 4:**
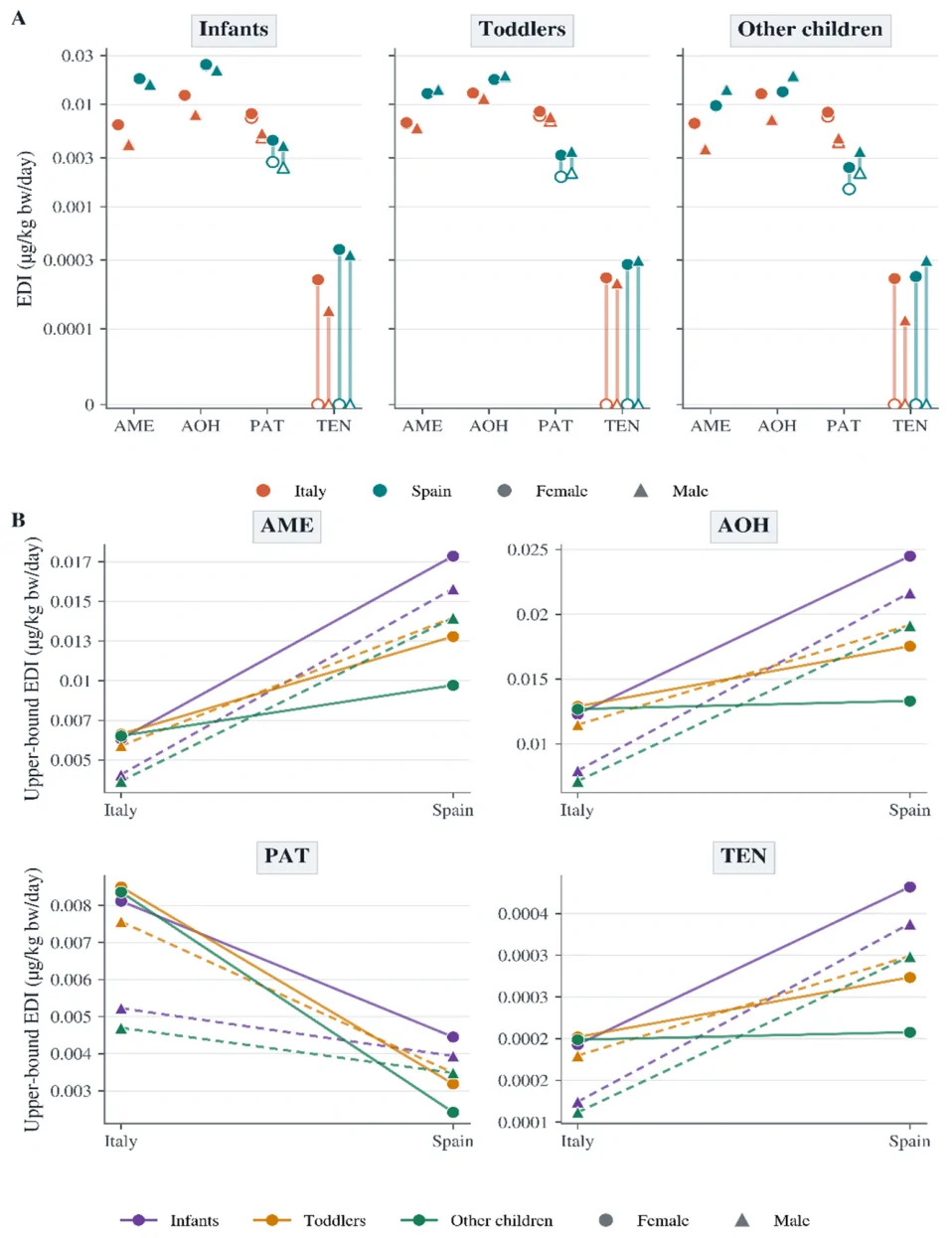
Country-specific chronic exposure to AME, AOH, PAT and TEN. (**A**) LB–UB EDI point-range estimates by age group, country and sex; open points show LB and solid symbols show UB estimates. (**B**) Italy–Spain slope comparison of UB EDI, with each line connecting matched age–sex strata.

**Figure 5:**
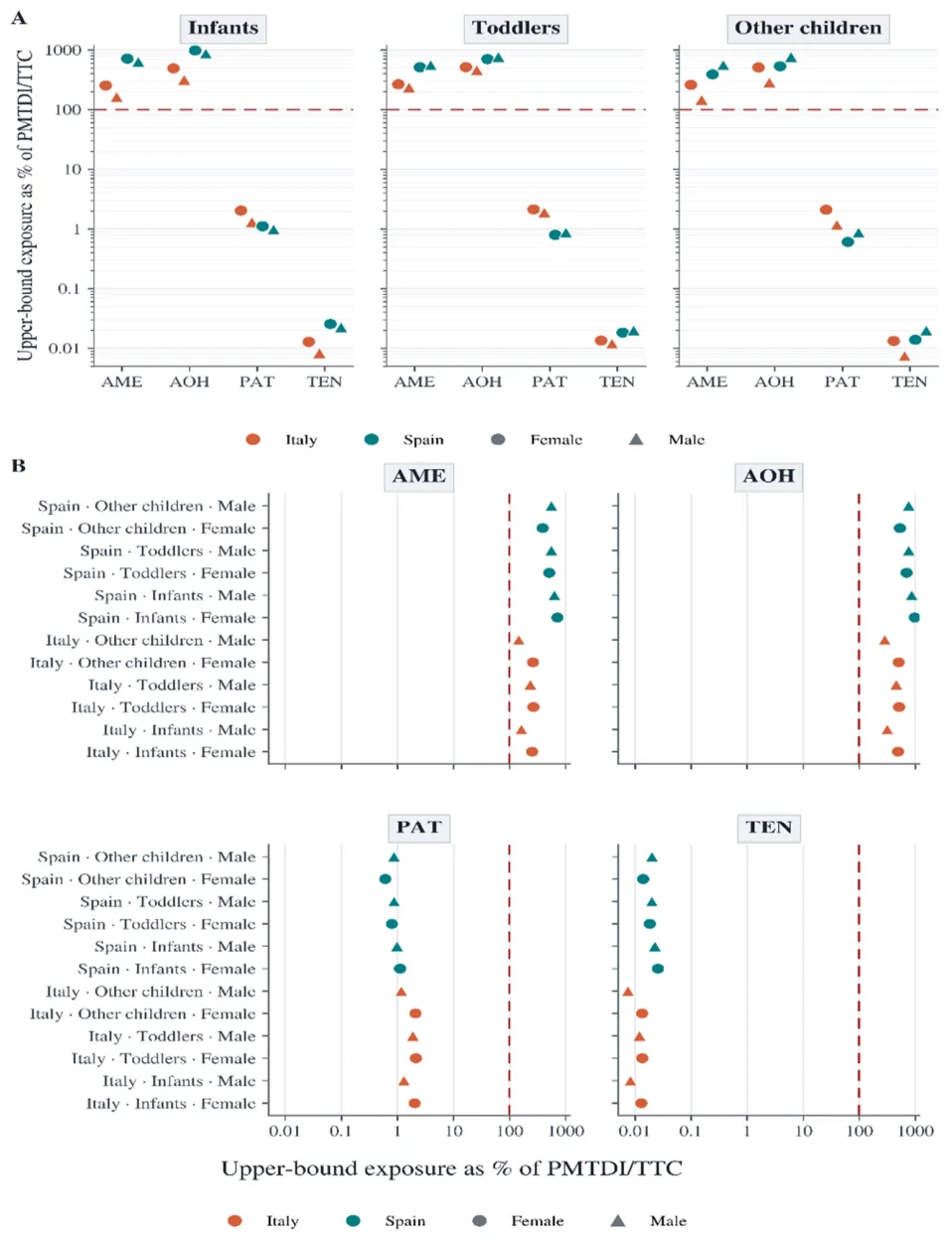
Upper-bound risk characterization. (**A**) UB exposure as a percentage of the applicable PMTDI or TTC by toxin, age group, country and sex; the red dashed line marks 100%. (**B**) Population-level UB risk distribution for all country–age–sex strata on a logarithmic scale.

**Figure 6:**
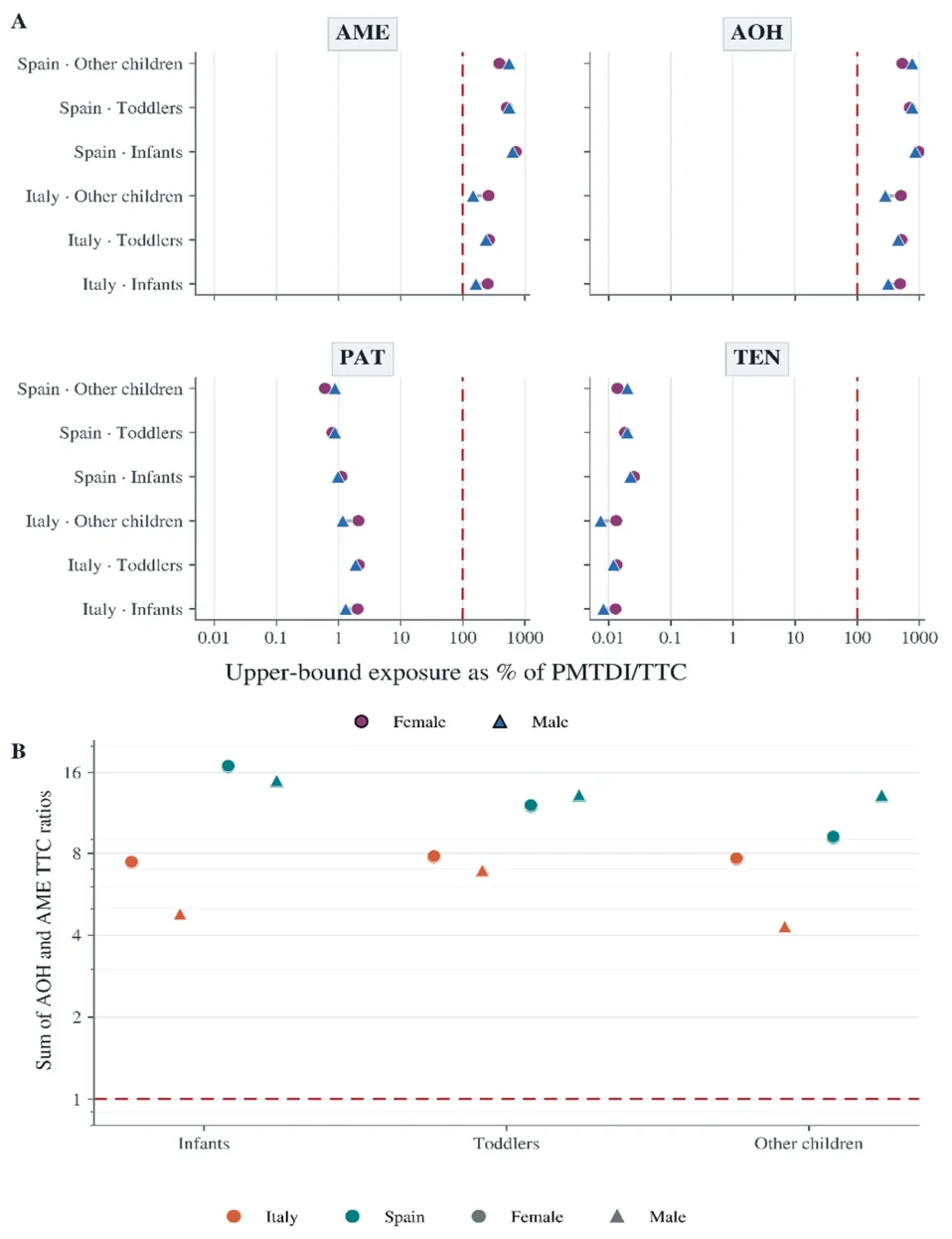
Sex-specific risk patterns and cumulative screening. (**A**) Female–male dumbbell comparison of UB risk within each country and age group. (**B**) LB–UB sum of AOH and AME TTC ratios. The red line at 1 is a screening benchmark.

## Data Availability

The original contributions presented in this study are included in the article/Supplementary Materials. Further inquiries can be directed to the corresponding author.

## Supplementary Materials

The following supporting information can be downloaded at https://www.mdpi.com/article/10.3390/toxins18100413/s1, Table S1: Retention times and MRM parameters for the determination of the target mycotoxins; Table S2: Linearity, sensitivity and matrix effect of the method for the four target mycotoxins in fruit purée; Table S3: Recovery (%) of the target mycotoxins at three concentration levels (*n* = 6); Table S4: Intra-day and inter-day precision (RSD %, *n* = 6) at three concentration levels (5, 10, and 25 µg/kg); Table S5: Description of the fruit purée samples from the Italian market considered in this study (*n* = 20, from retail markets); Table S6: Description of the fruit purée samples from the Spanish market considered in this study (*n* = 20, from retail markets); Table S7: Co-occurrence of patulin (PAT) and *Alternaria* toxins (AOH, AME, TEN) in Italian fruit purée samples (*n* = 20); Table S8: Co-occurrence of patulin (PAT) and *Alternaria* toxins (AOH, AME, TEN) in Spanish fruit purée samples (*n* = 20); Table S9: Consumer-only mean consumption used in the exposure assessment (g/kg bw/day); Table S10: Country-specific occurrence data used for deterministic exposure assessment; Table S11: Estimated daily intake (EDI) and corresponding risk-characterization ratios for each country, age group, and sex; Table S12: Chemical structures, molecular formulas, monoisotopic masses, and database sources of the mycotoxins included in the suspect screening panel. Figure S1: Representative MRM chromatograms of PAT and *Alternaria* toxins on the selected HSS T3 C18 column; Figure S2: MRM chromatograms (196.1 → 112.1, ESI−) of tenuazonic acid (TeA) obtained on four different chromatographic columns: BEH C18, Zorbax Eclipse C8, Zorbax Eclipse C18, and HSS T3 C18; Figure S3: Optimization of Florisil amount (50–200 mg) used in the d-SPE clean-up step based on recovery (a) and matrix effect (b) values for each mycotoxin under study.
