# Supplementary material for: Occurrence and Dietary Exposure Assessment of Patulin and Major Alternaria Toxins in Commercial Infant Fruit Purées from Italian and Spanish Markets Using Targeted Analysis and Suspect Screening
Source: Toxins (Basel). 2026 Sep 24;18(10):413. doi: 10.3390/toxins18100413 (PMC13709402; doi:10.3390/toxins18100413)
Supplement: Supplementary file 1 [file toxins-18-00413-s001.zip › toxins-4510927-supplementary.pdf]

Table S1. Retention times and MRM parameters for the determination of the target mycotoxins.

Analyte	Retention time(min)	Molecular ion	Precursor ion (*m/z*)	Product ions (*m/z*)	Fragmentor (V)	CE (eV)
PAT	2.64	[M-H]-	153.0	109.1*/81.0/53.2	50	5/10/15
TeA	3.25	[M-H]-	196.1	139.0*/112.1/136.2	190	20/25/20
TEN	5.84	[M+H] +	415.2	312.1*/132.0/302.1	130	20/50/10
AOH	5.99	[M-H]-	257.0	215.0*/213.0/147.0	175	26/26/30
AME	7.48	[M-H]-	271.1	256.0*/228.0/213.0	150	20/27/40

Table S2. Linearity, sensitivity and matrix effect of the method for the four target mycotoxins in fruit purée.

Analyte	Calibration equation	R^2 (linearity)	LOD (μg/kg)	LOQ (μg/kg)	ME (%)	ML* (μg/kg)
PAT	y = 11.857x + 42.2	0.988	0.15	0.5	−77.11	10
AOH	y = 71.988x + 96.369	0.996	0.03	0.1	−35.09	—
AME	y = 521.75x + 240.4	0.998	0.03	0.1	−57.09	—
TEN	y = 288.2x + 724.57	0.99	0.03	0.1	−28.52	—

*ML=maximum limit.

Table S3. Recovery (%) of the target mycotoxins at three concentration levels (n = 6).

Analyte	5 μg/kg	10 μg/kg	25 μg/kg
PAT	112.2	110.1	90.8
AOH	107.1	84.1	80.4
AME	95.0	83.1	76.9
TEN	105.5	88.1	87.4

Table S4. Intra-day and inter-day precision (RSD %, n = 6) at three concentration levels (5, 10, and 25 μg/kg).

	Intra-day precision			Inter-day precision		
Analyte	5 μg/kg	10 μg/kg	25 μg/kg	5 μg/kg	10 μg/kg	25 μg/kg
PAT	14.73	4.52	5.08	18.54	15.95	7.20
AOH	6.00	2.71	2.36	18.66	2.60	2.74
AME	6.12	3.37	3.84	18.57	4.76	4.36
TEN	1.91	1.83	2.14	16.06	5.72	6.66

Table S5. Description of the fruit purée samples from the Italian market considered in this study (n = 20, from retail markets).

Sample Code	Composition	Apple Content (%)	Organic (1) Conventional (0)
ITA_1	Apple, Strawberry, Raspberry	81.7	0
ITA_2	Apple, Strawberry	89.9	0
ITA_3	Apple, Pear, Strawberry	60	1
ITA_4	Apple, Pear	50	0
ITA_5	Apple	100	0

Sample Code	Composition	Apple Content (%)	Organic (1) Conventional (0)
ITA_6	Banana, Apple	35	0
ITA_7	Apple, Peach, Mango	80	1
ITA_8	Apple	100	1
ITA_9	Apple, Peach, Apricot	75	1
ITA_10	Apple, Red fruits	70	1
ITA_11	Apple	100	1
ITA_12	Apple, Banana, Strawberry	60	1
ITA_13	Apple	100	0
ITA_14	Apple, Blueberry	87.9	0
ITA_15	Apple, Pear, Banana	53.9	0
ITA_16	Apple, Carrot, Apricot	80	1
ITA_17	Pear, Plum, Black currant	-	1
ITA_18	Apple, Pear	50	1
ITA_19	Apple, Strawberry, Plantain, Blueberry	58	0
ITA_20	Apple, Pear, Banana	53.9	0

Table S6. Description of the fruit purée samples from the Spanish market considered in this study (n = 20, from retail markets).

Sample Code	Composition	Apple Content (%)	Organic (1) Conventional (0)
ESP_1	Multi fruits	88	1
ESP_2	Apple, Mango	80	1
ESP_3	Apple, Strawberries	90	1
ESP_4	Apple, Banana	89	1
ESP_5	Apple, Pear, Apricot	89	1
ESP_6	Apple, Banana	70	1
ESP_7	Apple, Banana	69	0
ESP_8	Apple, Strawberry, Banana	58	1
ESP_9	Apple, Peach, Banana	55	1
ESP_10	Apple, Banana, Yogurt	49	1
ESP_11	Apple, Pear	45	1
ESP_12	Banana, Apple	46	0
ESP_13	Apple, Pear	44	0
ESP_14	Banana, Strawberry, Apple	22	0
ESP_15	Apple	100	1
ESP_16	Pear	-	1
ESP_17	Apple, Strawberry, Banana	57	0
ESP_18	Apple, Pear, Peach, Banana	70	0
ESP_19	Banana, Apple, Strawberry	42	0

Sample Code	Composition	Apple Content (%)	Organic (1) Conventional (0)
ESP_20	Apple, Pear, Peach	80	0

Table S7. Co-occurrence of patulin (PAT) and *Alternaria* toxins (AOH, AME, TEN) in Italian fruit purée samples (n = 20).

Sample Code	PAT	AOH	AME	TEN
ITA_1	ND	8.67 ± 0.18	4.60 ± 0.58	ND
ITA_2	ND	7.67 ± 0.10	4.17 ± 0.07	ND
ITA_3	<LOQ	0.29 ± 0.19	0.38 ± 0.17	ND
ITA_4	11.60 ± 1.22	0.83 ± 0.13	0.30 ± 0.16	ND
ITA_5	ND	1.58 ± 0.02	0.69 ± 0.28	ND
ITA_6	ND	ND	ND	ND
ITA_7	ND	ND	ND	ND
ITA_8	1.33 ± 0.60	ND	0.11 ± 0.06	ND
ITA_9	ND	1.20 ± 0.45	0.43	ND
ITA_10	2.32 ± 0.21	4.04 ± 0.59	3.41 ± 0.60	ND
ITA_11	1.16	8.38 ± 0.97	3.09 ± 0.73	ND
ITA_12	ND	ND	ND	ND
ITA_13	ND	1.18 ± 0.11	1.25 ± 0.50	ND
ITA_14	6.58 ± 0.75	<LOQ	0.28 ± 0.04	ND
ITA_15	ND	1.49 ± 0.14	0.89 ± 0.04	ND
ITA_16	ND	0.25	<LOQ	ND
ITA_17	ND	0.69 ± 0.03	0.45 ± 0.10	ND
ITA_18	ND	0.75 ± 0.13	0.79 ± 0.01	ND
ITA_19	ND	ND	ND	ND
ITA_20	ND	1.06 ± 0.64	0.62 ± 0.65	ND

Concentrations expressed as μg/kg (mean ± SD, n = 4). ND: not detected: <LOQ: below the limit of quantification.

Table S8. Co-occurrence of patulin (PAT) and *Alternaria* toxins (AOH, AME, TEN) in Spanish fruit purée samples (n = 20).

Sample Code	PAT	AOH	AME	TEN
ESP_1	ND	4.22 ± 0.09	3.63 ± 0.30	ND
ESP_2	ND	ND	<LOQ	ND
ESP_3	ND	6.92 ± 0.42	2.64 ± 0.19	ND
ESP_4	ND	2.90 ± 0.78	2.35 ± 0.22	ND
ESP_5	2.81	5.99 ± 0.01	4.78 ± 0.52	ND
ESP_6	ND	ND	ND	ND
ESP_7	ND	ND	ND	ND
ESP_8	ND	ND	0.18 ±0.09	ND

Sample Code	PAT	AOH	AME	TEN
ESP_9	ND	0.1 (LOQ)	0.14 ± 0.09	ND
ESP_10	ND	ND	ND	ND
ESP_11	ND	1.26 ± 0.35	0.36 ± 0.00	ND
ESP_12	ND	ND	0.12 ± 0.07	ND
ESP_13	ND	3.12 ± 0.13	1.44 ± 0.14	ND
ESP_14	ND	3.86 ± 0.09	5.90 ± 0.01	ND
ESP_15	ND	ND	ND	ND
ESP_16	ND	1.78 ± 0.11	1.47 ± 0.20	ND
ESP_17	ND	1.37 ± 0.16	1.59 ± 0.18	ND
ESP_18	1.48 ± 0.86	2.61 ± 0.35	1.86 ± 0.12	ND
ESP_19	ND	ND	ND	ND
ESP_20	ND	4.09 ± 0.65	1.39 ± 0.15	ND

Concentrations expressed as µg/kg (mean ± SD, n = 4). ND: not detected: <LOQ: below the limit of quantification.

Table S9. Consumer-only mean consumption used in the exposure assessment (g/kg bw/day).

Country	Age group	Female	Male
Italy	Infants	6.430	4.141
Italy	Toddlers	6.733	5.992
Italy	Other children	6.624	3.722
Spain	Infants	12.733	11.265
Spain	Toddlers	9.118	9.967
Spain	Other children	6.923	9.953

Table S10. Country-specific occurrence data used for deterministic exposure assessment.

Country	Toxin	Numeric n/20	ND/<LOQ	Mean LB (µg/kg)	Mean UB (µg/kg)	Max numeric (µg/kg)
Italy	AME	15	4/1	0.9780	0.9840	4.60
Italy	AOH	14	5/1	1.9070	1.915	8.67
Italy	PAT	5	15/0	1.1495	1.2795	11.6
Italy	TEN	0	20/0	0.0000	0.0300	—
Spain	AME	14	5/1	1.3950	1.4025	5.90
Spain	AOH	12	8/0	1.9110	1.9230	6.92
Spain	PAT	2	18/0	0.2145	0.3495	2.81
Spain	TEN	0	20/0	0.0000	0.0300	—

ND values were replaced by 0 (LB) or the LOD (UB); explicit <LOQ results were replaced by 0 (LB) or the LOQ (UB). Reported numeric results were retained as reported, including values below the nominal LOQ. ND/<LOQ gives the number of non-detects and explicit <LOQ results, respectively

Table S11. Estimated daily intake (EDI) and corresponding risk-characterization ratios for each country, age group, and sex.

Country	Age group	Sex	Toxin	EDI, μg/kg bw/day		Risk metric	Risk metric value	
				LB	UB		LB	UB
Italy	Infants	F	PAT	0.00681	0.00823	HQ	0.0170	0.0206
Italy	Infants	F	AOH	0.0123	0.0123	EDI/TTC	4.91	4.92
Italy	Infants	F	AME	0.00629	0.00633	EDI/TTC	2.52	2.53
Italy	Infants	F	TEN	0	0.000193	EDI/TTC	0	0.000129
Italy	Infants	M	PAT	0.00439	0.00530	HQ	0.0110	0.0132
Italy	Infants	M	AOH	0.00790	0.00793	EDI/TTC	3.16	3.17
Italy	Infants	M	AME	0.00405	0.00408	EDI/TTC	1.62	1.63
Italy	Infants	M	TEN	0	0.000124	EDI/TTC	0	0.0000830
Italy	Toddlers	F	PAT	0.00713	0.00861	HQ	0.0178	0.0215
Italy	Toddlers	F	AOH	0.0128	0.0129	EDI/TTC	5.14	5.16
Italy	Toddlers	F	AME	0.00659	0.00663	EDI/TTC	2.63	2.65
Italy	Toddlers	F	TEN	0	0.000202	EDI/TTC	0	0.000135
Italy	Toddlers	M	PAT	0.00635	0.00767	HQ	0.0159	0.0192
Italy	Toddlers	M	AOH	0.0114	0.0115	EDI/TTC	4.57	4.59
Italy	Toddlers	M	AME	0.00586	0.00590	EDI/TTC	2.34	2.36
Italy	Toddlers	M	TEN	0	0.000180	EDI/TTC	0	0.000120
Italy	Other children	F	PAT	0.00702	0.00848	HQ	0.0175	0.0212
Italy	Other children	F	AOH	0.0126	0.0127	EDI/TTC	5.05	5.07
Italy	Other children	F	AME	0.00648	0.00652	EDI/TTC	2.59	2.61
Italy	Other children	F	TEN	0	0.000199	EDI/TTC	0	0.000132
Italy	Other children	M	PAT	0.00394	0.00476	HQ	0.00990	0.0119
Italy	Other children	M	AOH	0.00710	0.00713	EDI/TTC	2.84	2.85
Italy	Other children	M	AME	0.00364	0.00366	EDI/TTC	1.46	1.47
Italy	Other children	M	TEN	0	0.000112	EDI/TTC	0	0.0000740
Spain	Infants	F	PAT	0.00273	0.00445	HQ	0.00680	0.0111
Spain	Infants	F	AOH	0.0243	0.0245	EDI/TTC	9.73	9.79
Spain	Infants	F	AME	0.0178	0.0179	EDI/TTC	7.11	7.14
Spain	Infants	F	TEN	0	0.000382	EDI/TTC	0	0.000255
Spain	Infants	M	PAT	0.00242	0.00394	HQ	0.00604	0.00984
Spain	Infants	M	AOH	0.0215	0.0217	EDI/TTC	8.61	8.67

Country	Age group	Sex	Toxin	EDI, µg/kg bw/day		Risk metric	Risk metric value	
				LB	UB		LB	UB
Spain	Infants	M	AME	0.0157	0.0158	EDI/TTC	6.29	6.32
Spain	Infants	M	TEN	0	0.000338	EDI/TTC	0	0.000225
Spain	Toddlers	F	PAT	0.00196	0.00319	HQ	0.00489	0.00797
Spain	Toddlers	F	AOH	0.0174	0.0175	EDI/TTC	6.97	7.01
Spain	Toddlers	F	AME	0.0127	0.0128	EDI/TTC	5.09	5.12
Spain	Toddlers	F	TEN	0	0.000274	EDI/TTC	0	0.000182
Spain	Toddlers	M	PAT	0.00214	0.00348	HQ	0.00535	0.00871
Spain	Toddlers	M	AOH	0.0190	0.0192	EDI/TTC	7.62	7.67
Spain	Toddlers	M	AME	0.0139	0.0140	EDI/TTC	5.56	5.59
Spain	Toddlers	M	TEN	0	0.000299	EDI/TTC	0	0.000199
Spain	Other children	F	PAT	0.00149	0.00242	HQ	0.00371	0.00605
Spain	Other children	F	AOH	0.0132	0.0133	EDI/TTC	5.29	5.33
Spain	Other children	F	AME	0.00966	0.00971	EDI/TTC	3.86	3.88
Spain	Other children	F	TEN	0	0.000208	EDI/TTC	0	0.000138
Spain	Other children	M	PAT	0.00214	0.00348	HQ	0.00534	0.00870
Spain	Other children	M	AOH	0.0190	0.0191	EDI/TTC	7.61	7.66
Spain	Other children	M	AME	0.0139	0.0140	EDI/TTC	5.55	5.58
Spain	Other children	M	TEN	0	0.000299	EDI/TTC	0	0.000199

EDI: estimated daily intake, expressed as µg/kg bw/day. For PAT, HQ = EDI/PMTDI (PMTDI = 0.4 µg/kg bw/day). For AOH, AME and TEN, ratio = EDI/TTC (TTC = 0.0025, 0.0025 and 1.5 µg/kg bw/day, respectively). LB: lower bound; UB: upper bound.

Table S12. Chemical structures, molecular formulas, monoisotopic masses, and database sources of the mycotoxins included in the suspect screening panel.

Structure	Name	Formula	Monoisotopic mass	Source
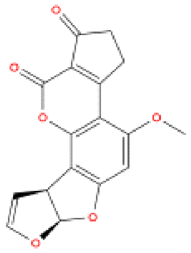	Aflatoxin B1	C17H12O6	312.0633	Chemspider
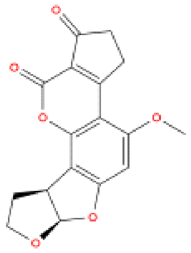	Aflatoxin B2	C17H14O6	314.079	Chemspider
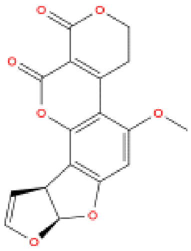	Aflatoxin G1	C17H12O7	328.0583	Chemspider
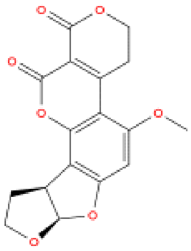	Aflatoxin G2	C17H14O7	330.0739	Chemspider
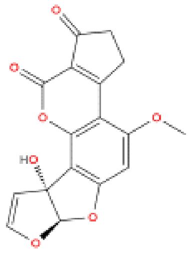	Aflatoxin M1	C17H12O7	328.0583	Chemspider

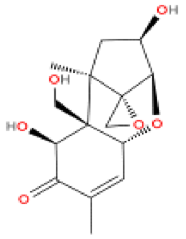	Deoxynivalenol	C15H20O6	296.1259	Chemspider
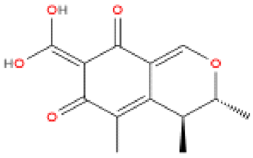	Citrinin	C13H14O5	250.0841	Chemspider
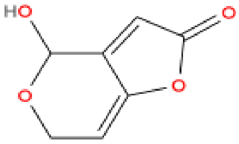	Patulin	C7H6O4	154.0266	Chemspider
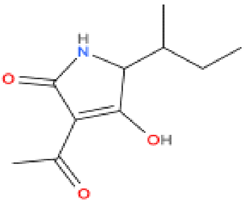	Tenuazonic acid	C10H15NO3	197.1051	Chemspider
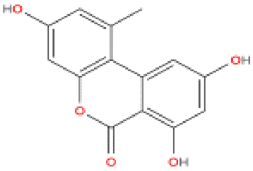	Alternariol	C14H10O5	258.0528	Chemspider
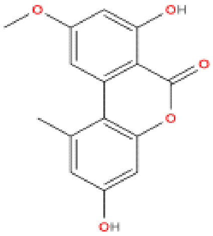	Alternariol mono methyl ether	C15H12O5	272.0684	Pubchem

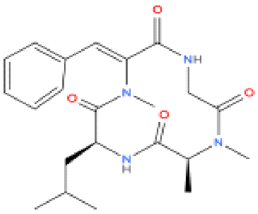	Tentoxin	$C_{22}H_{30}N_4O_4$	414.2267	Chemspider
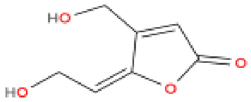	(E)-ascladiol	$C_7H_8O_4$	156.0422	Chemspider
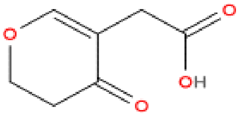	Deoxypatulinic acid	$C_7H_8O_4$	156.0422	Chemspider
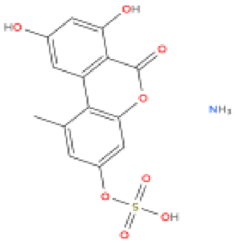	Alternariol 3 - sulfate ammonium salt	$C_{14}H_{13}NO_8S$	355.0361	Pubchem
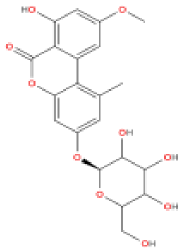	3-O-β-D-Glucopyranosyl Alternariol	$C_{20}H_{20}O_{10}$	420.106 (accurate mass)	LGC standards
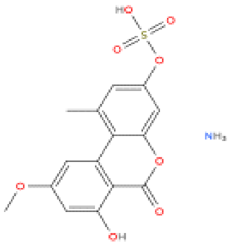	Alternariol 9-methyl ether 3-sulfate (ammonium salt)	$C_{15}H_{15}NO_8S$	369.06 (accurate mass)	LGC standards

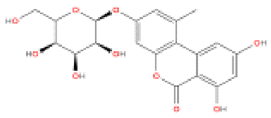	3-O-β-D-Glucopyranosyl Alternariol-9-methyl Ether	C21H22O10	434.121 (accurate mass)	LGC standards
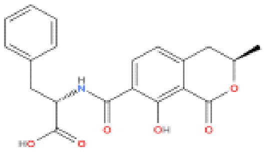	Ochratoxin B	C20H19NO6	369.1212	Chemspider
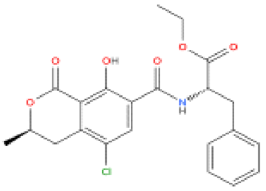	Ochratoxin C	C22H22ClNO6	431.1135	Chemspider
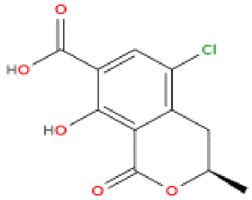	Ochratoxin alpha	C11H9ClO5	256.0138	Chemspider
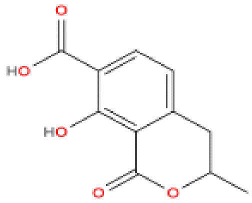	Ochratoxin β	C11H10O5	222.0528	Chemspider
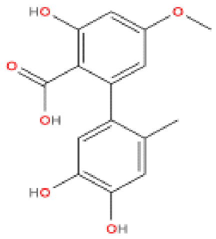	Altenusin	C15H14O6	290.079	Chemspider

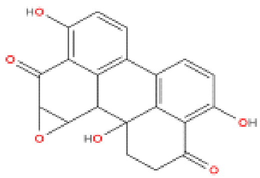	Altertoxin-II	C20H14O6	350.079	Pubchem
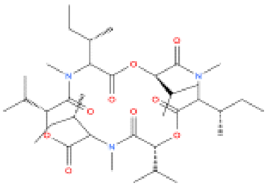	Enniatin A	C36H63N3O9	681.4564	Chemspider
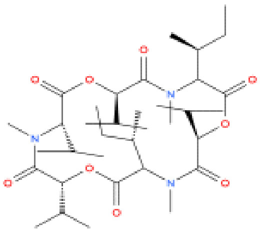	Enniatin A1	C35H61N3O9	667.4407	Chemspider
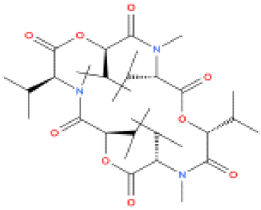	Enniatin B	C33H57N3O9	639.4094	Chemspider
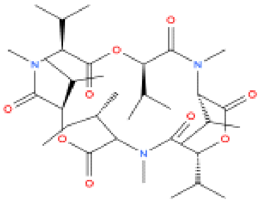	Enniatin B1	C34H59N3O9	653.4251	Chemspider
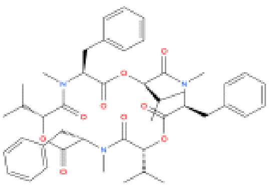	Beauvericin	C45H57N3O9	783.4094	Chemspider

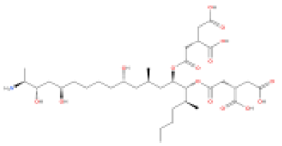	Fumonisin B1	$C_{34}H_{59}NO_{15}$	721.388	Chemspider
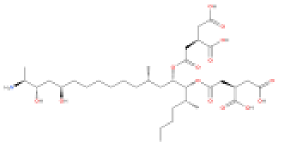	Fumonisin B2	$C_{34}H_{59}NO_{14}$	705.3935	Chemspider
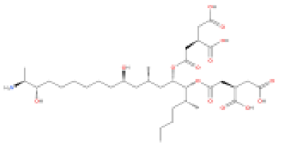	Fumonisin B3	$C_{34}H_{59}NO_{14}$	705.3935	Chemspider
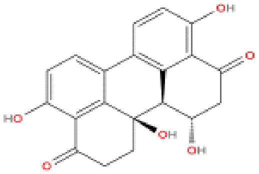	Altertoxin I	$C_{20}H_{16}O_6$	352.0946	Chemspider
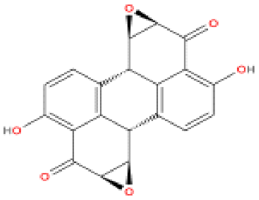	Altertoxin III	$C_{20}H_{12}O_6$	348.0633	Pubchem
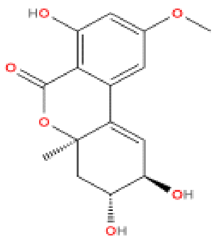	Altenuene	$C_{15}H_{16}O_6$	292.0946	Chemspider

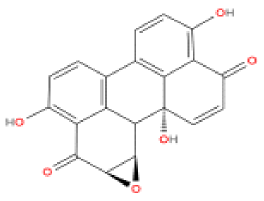	Stemphyltoxin III	C20H12O6	348.0633	Pubchem
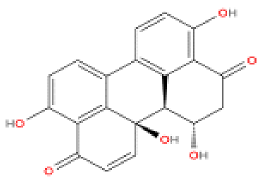	Alterperylenol	C20H14O6	350.079	Chemspider
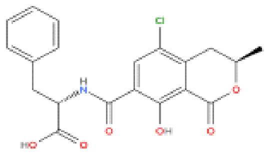	Ochratoxin A	C20H18ClNO6	403.0822	Chemspider
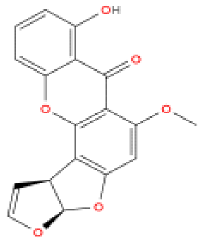	Sterigmatocystin	C18H12O6	324.0633	Chemspider
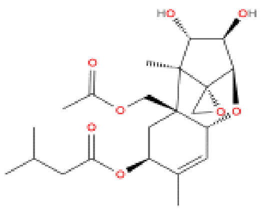	HT-2	C22H32O8	424.209	Chemspider
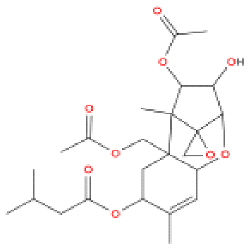	T-2	C24H34O9	466.22	Chemspider

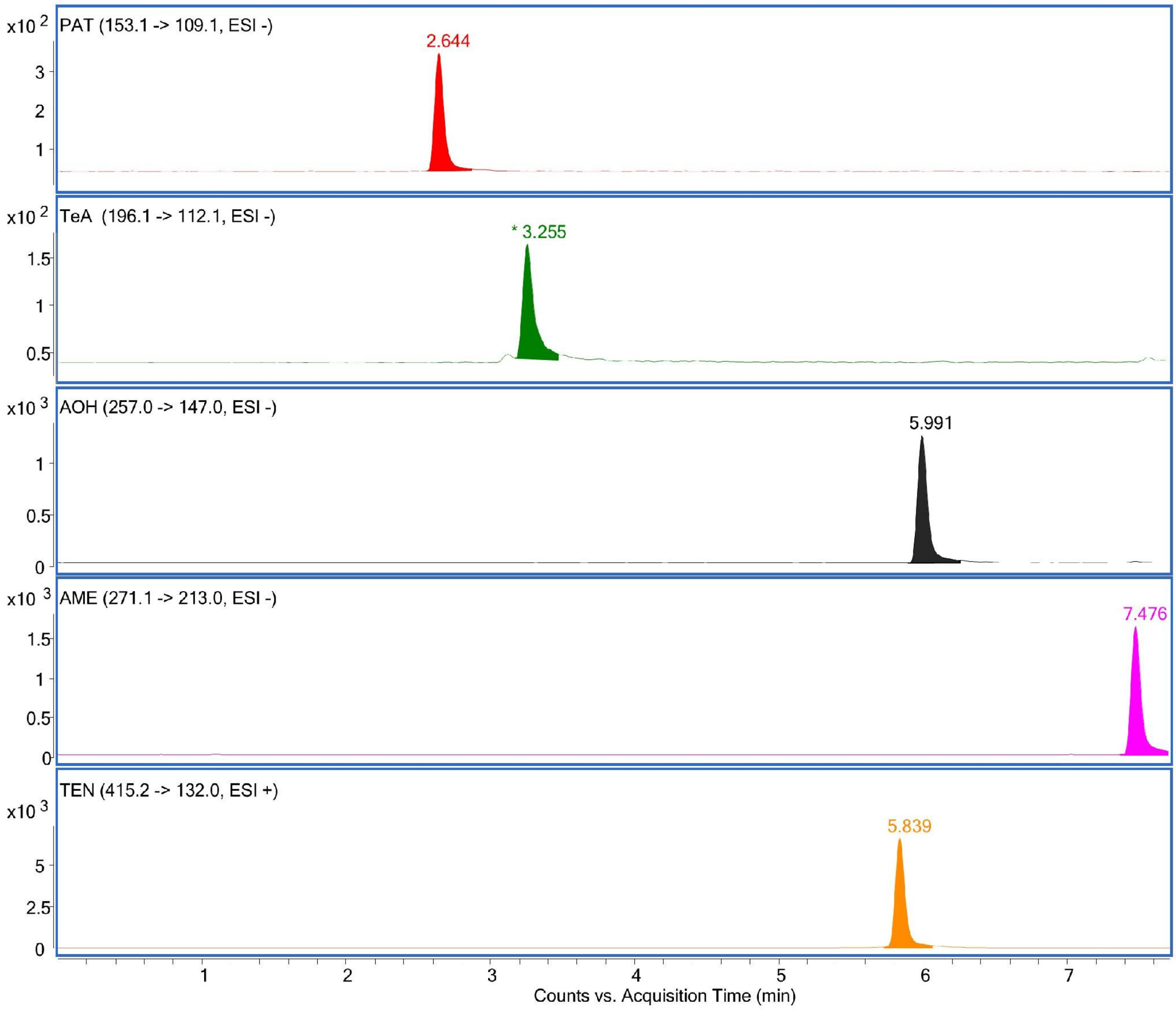

Figure S1. Representative MRM chromatograms of PAT and *Alternaria* toxins on the selected HSS T3 C18 column

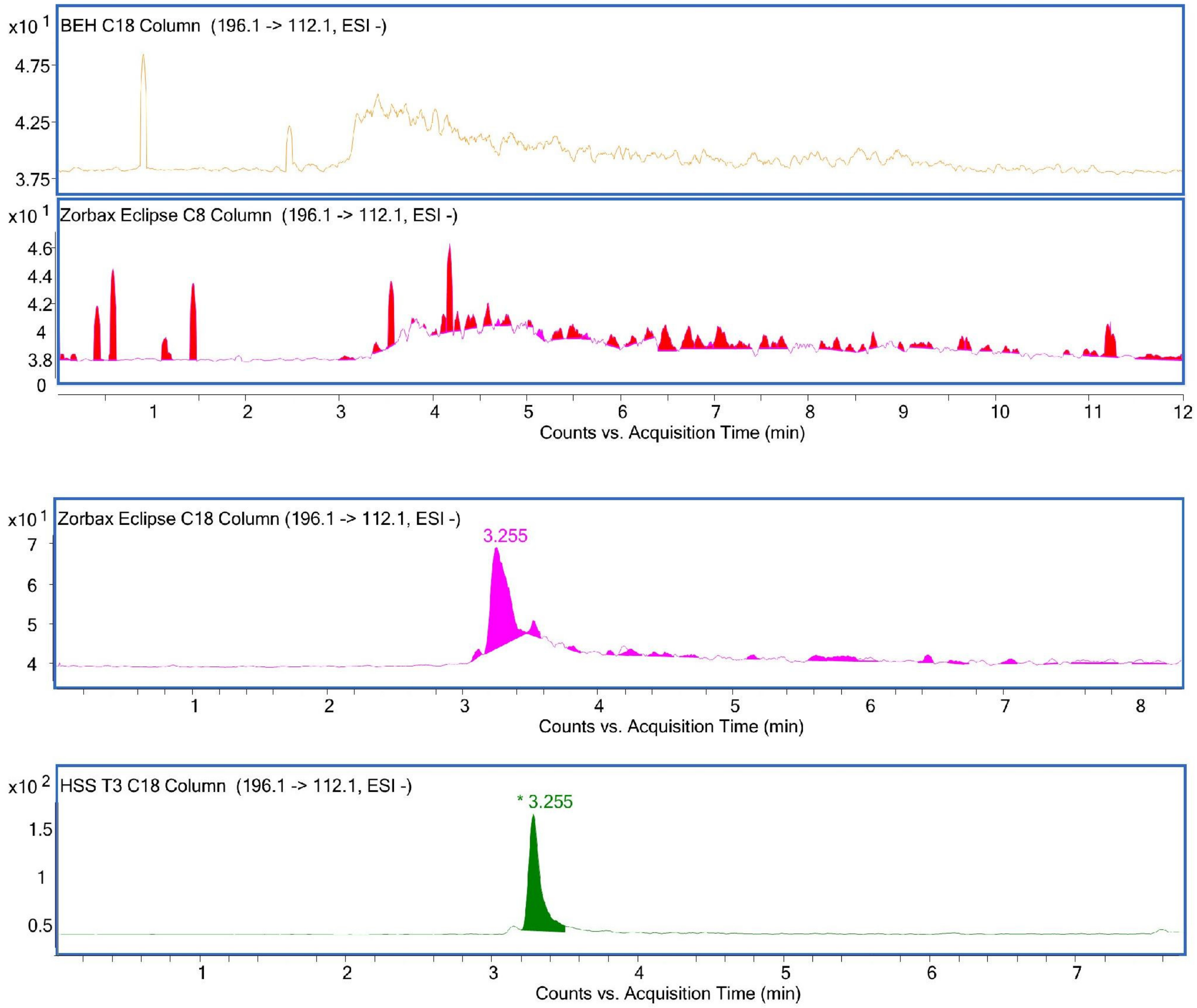

Figure S2. MRM chromatograms (196.1 → 112.1, ESI−) of tenuazonic acid (TeA) obtained on four different chromatographic columns: BEH C18, Zorbax Eclipse C8, Zorbax Eclipse C18, and HSS T3 C18.

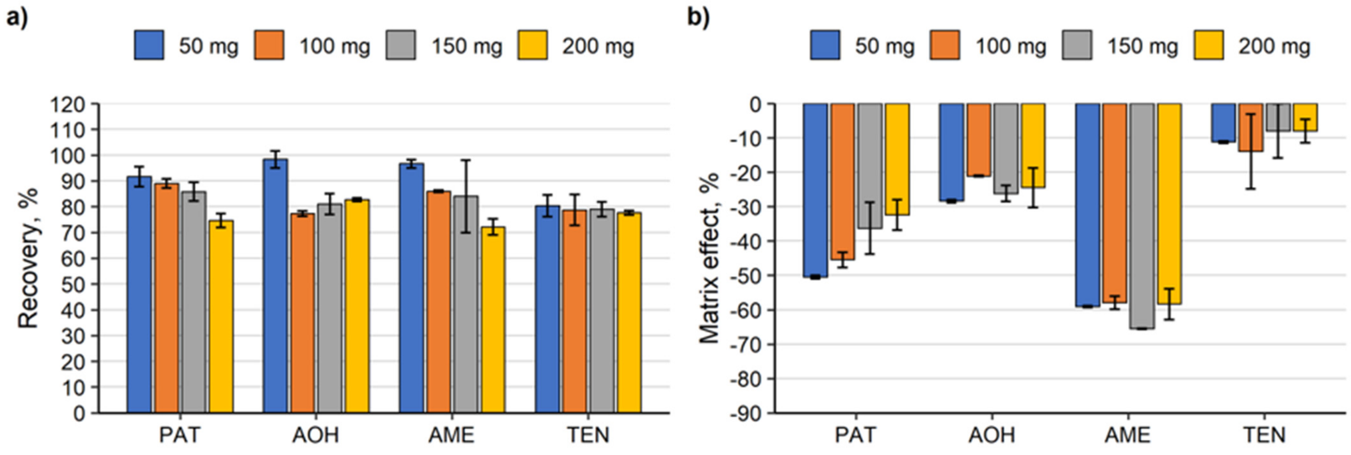

Figure S3. Optimization of Florisil amount (50-200 mg) used in the d-SPE clean-up step based on recovery (a) and matrix effect (b) values for each mycotoxin under study.
